# Can we add another C to the 6Cs: C for Clever?

**DOI:** 10.1002/nop2.437

**Published:** 2019-12-18

**Authors:** Parveen Ali

**Affiliations:** ^1^ Division of Nursing and Midwifery Health Sciences School The University of Sheffield Sheffield UK

“You are so clever, why are you doing Nursing?”

“You could easily be a doctor, so don't waste yourself in Nursing?”

“Oh, so you are a nurse? You are very intelligent, and I don't understand why you decided to become a nurse?”

“When are you going to be a doctor?” “To become a nurse, you need a good heart, a caring personality and humility.”

“you do need to be obedient!”

Since I joined the nursing profession, in the 1990s, these are some of the statements that I have heard from many well‐wishers, friends and those who were really concerned about me or those who were impressed with my progress, ability and work. Sometimes, these people were senior nurses and colleagues. Sadly, things have not changed in over 20 years. You would think that people know more about nursing now. But this is not the case. People still do not know much about nursing. We still have not managed to change or improve the image of nursing to the general public. This has implications for future generation of nursing students who do not fully understand the nature of the profession and what it requires to be a nurse. I strongly believe that we, nurses, have a responsibility to articulate clearly what nursing is all about and whether nurses need to be intelligent and educated in addition to being a caring, compassionate and a nice person. In this piece, I explain what made me reflect on this issue and why we need to state clearly that nurses need to be intelligent.

The nursing profession has developed over the past few decades and now nurses perform many complex roles in various healthcare settings. Nurses care for critically ill people in intensive care units, severely injured or those with acute care needs in emergency departments and those undergoing surgery in the operation theatre (Gaffney, Hatcher, & Milligan, 2016; Niu, Li, Tang, Gong, & Zhang, 2017). Nurses help people with long‐term conditions manage their symptoms in their home by providing outpatient and community care services. Nurses work as autonomous practitioners who are able to think critically and make complex and timely clinical decisions to provide an appropriate level of care to their clients, including being able to prescribe across the whole range of the pharmacopeia. Therefore, they need to have the right ability, education and skill to effectively perform these complex roles (Ali & Watson, 2011). However, this is not always evident to the general public, including those wanting to become a nurse. As a nurse educator, one of my responsibilities is to interview potential nursing students for the pre‐registration nursing programmes to ensure that we only select candidates who we think can become caring, competent and effective nurses at the end of their educational programme. In such interviews, candidates are asked to talk about their understanding of the nursing profession and essential attributes of the nurses. Most candidates talk about ‘6 Cs’, namely care; compassion; competence; communication; courage; and commitment (Darbyshire & McKenna, 2013). On probing further, most will also mention that nurses need to be selfless and that they need to put their patients first. I have never heard any candidate mentioning that nurses need to be intelligent, or nurses need to be creative or nurses need to be able to make quick and timely decisions. Recently, when interviewing, I asked a potential candidate if a nurse needs to be intelligent. The candidate got confused, took a few seconds to think and said “no, a nurse doesn't need to be intelligent” as she just need to follow orders, so she needs to be obedient more than intelligent. This was hardly a surprise. But what does it actually tell us? What do we need to learn from it?

It tells me that the information out there about nurses and the nursing profession is not accurate. It tells me that perhaps as nurses, we do not talk about intelligence, critical thinking, creativity and accountability as essential attributes of a nurse. Following an Internet search, it was not easy to find information about intelligence in relation to nursing. Young people aspiring to be nurses search the Internet to find out about nursing and use that information to develop their understanding of nursing and present the same information in interviews.

Most candidates mention the 6Cs as values underpinning nursing or essential attributes of a nurse. The 6Cs, proposed by the then Chief Nursing Officer for England, Jane Cummings, and Department of Health Nursing Director, Viv Bennett (Department of Health, 2012), are considered key areas for action and behaviours that underpin good nursing practice. The 6Cs are considered applicable to every healthcare worker; however, it is frequently referred to as a nursing concept and: ‘… fundamental in nursing care’ (Foulds, Timms, Barwell, & Gunning, 2015, p. S4). While the 6Cs is a powerful concept and suggests values that underpin practice, it does not capture what it requires for nurses to develop these attributes. To me at least, the 6Cs perpetuates the idea that nurses only need the softer and essentially inherent characteristics which do not require any education or preparation. There is no doubt that the 6Cs has a considerable impact and we also know that nurses not only need these attributes, but also require critical thinking, decision‐making and leadership skills. However, if we must have the 6 Cs, it is possible to add at least one other ‘C’ for ‘Clever’ and if it will make a difference. It may make aspiring nursing students think that nurses need to be intelligent and clever and force them to think why?

The responsibility lies with us to ensure that when we talk about how care, compassion, competence, communication, courage, commitment is important, we also highlight that to be a nurse one need to be intelligent and educated, as they need to act as an advocate for individuals, families and communities whom they serve, to make critical decisions in the delivery of care, in the management of the healthcare system and in developing health‐related research.
